# In this issue: Volume 115, Issue 1, January 2024

**DOI:** 10.1111/cas.16071

**Published:** 2024-01-28

## Extracellular vesicle‐encapsulated microRNA‐296‐3p from cancer‐associated fibroblasts promotes ovarian cancer development through regulation of the PTEN/AKT and SOCS6/STAT3 pathways

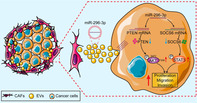

Ovarian cancer is a highly lethal form of cancer that affects women worldwide. However, our understanding of its progression and treatment options is limited. Cancer progression involves the release of tiny particles called extracellular vesicles (EVs) by specific cells known as cancer‐associated fibroblasts (CAFs). These particles are important components of the tumor microenvironment and might have an important role in promoting cancer spread and growth within the body. These vesicles can carry molecules like miRNAs, such as miR‐296‐3p, which has been found to influence how cancer cells multiply, move, and resist drugs. Although recent discoveries have demonstrated that miR‐296‐3p regulates distinct genes in various cancers to either promote or inhibit tumor growth, its function in ovarian cancer is still unclear.

To explore this aspect, researchers investigated whether EVs loaded with miR‐296‐3p play a significant role in the development and spread of ovarian cancer. They found that when miR‐296‐3p is overproduced in these EVs from CAFs, it significantly boosts the abilities of ovarian cancer cells to multiply, invade other tissues, and resist drugs used in treatment. Further investigation revealed that miR‐296‐3p achieves this by directly affecting certain genes (PTEN and SOCS6) and activating specific cellular pathways (AKT and STAT3) linked to cancer progression.

The researchers noted that higher levels of miR‐296‐3p found in EVs had a close link with tumor formation in ovarian cancer patients. Moreover, these patients showed resistance to standard ovarian cancer treatments. This suggests that miR‐296‐3p within these EVs could potentially serve as a marker for diagnosing ovarian cancer and serve as a promising target for new treatments.

Altogether, these findings shed light on how CAF‐derived EVs carrying miR‐296‐3p contribute to ovarian cancer's aggressive nature. The research has highlighted their potential as both a diagnostic biomarker and a target for developing future therapeutics for cancer treatments.

https://onlinelibrary.wiley.com/doi/full/10.1111/CAS.16014

## SCD1 inhibition enhances the effector functions of CD8+ T cells via ACAT1‐dependent reduction of esterified cholesterol

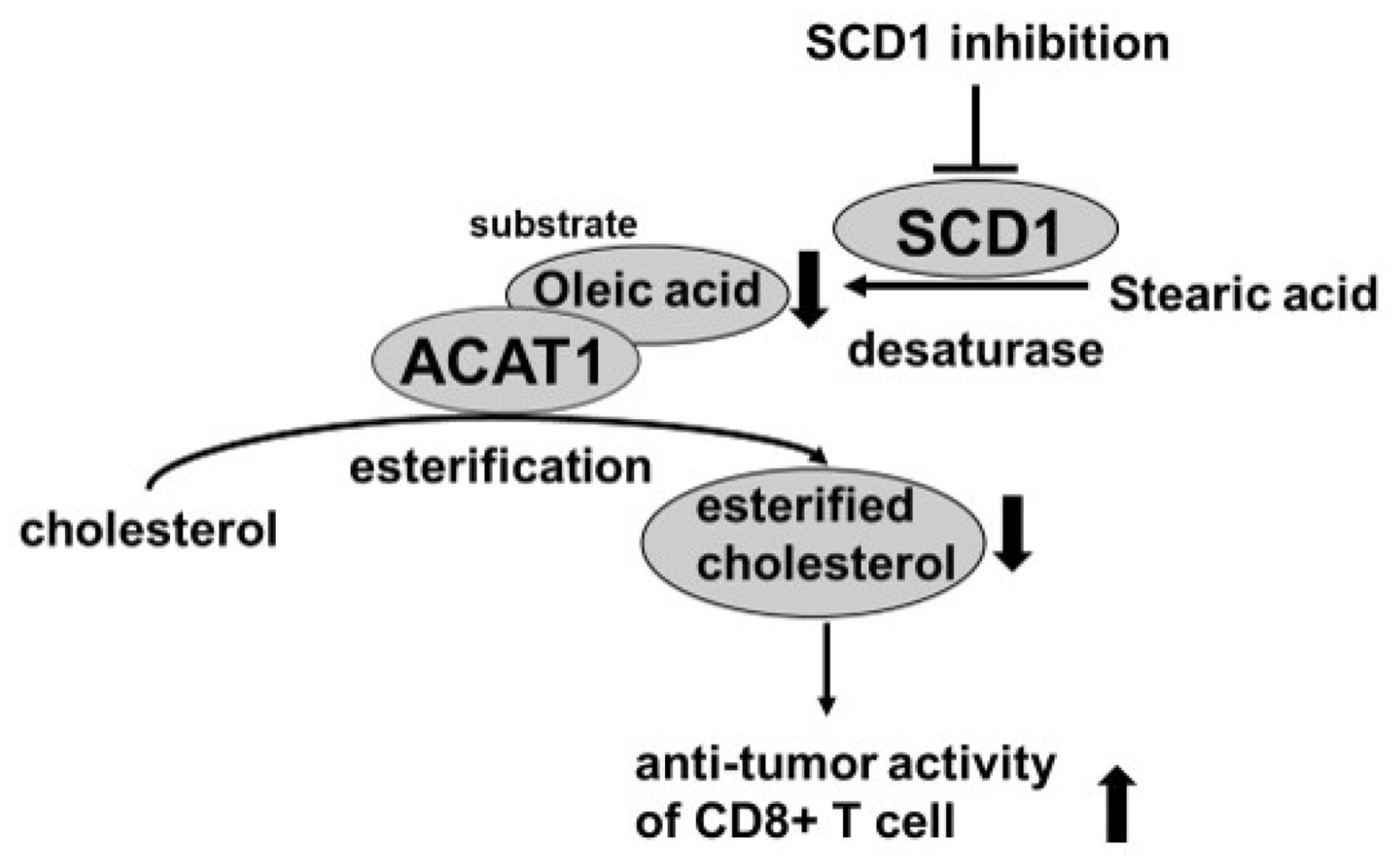

Immunotherapy is a type of cancer treatment that uses the body's own immune system to fight cancer. It is less toxic compared to other cancer treatments. However, it is not effective on all cancer patients and its effects depend on the immune environment around the tumor. Therefore, it is important to study and develop new immunotherapy strategies.

Previous studies have shown that inhibition of the enzymes stearoyl‐CoA desaturase 1 (SCD1) and acetyl‐CoA acetyltransferase 1 (ACAT1) improves the immune system's ability to fight cancer. These enzymes play an important role in fat and cholesterol metabolism, respectively. The enzyme SCD1 helps in production of a fatty acid called oleic acid and ACATI converts oleic acid to esterified cholesterol. These enzymes are known to significantly influence immune cell performance. However, the underlying process through which inhibitors of these enzymes enhance the function of immune cells is unknown.

Researchers have now identified the relation between SCD1 and ACAT1 and the novel mechanism through which the inhibitors of these enzymes improve the function of CD8^+^ T cells. CD8^+^ T cells are a type of immune cells that produce chemicals called cytokines to eliminate tumor cells.

The researchers studied the effects of enzyme inhibitors on lab‐cultured cells and in tumor‐bearing mice. They observed that inhibition of SCD1 resulted in decrease in oleic acid and esterified cholesterol produced by ACAT1. This in turn enhanced the ability of CD8^+^ T cells to destroy cancer cells due to increased production of a cytokine called interferon gamma (INF‐γ).

These findings indicate that SCD1 inhibitors and ACAT1 inhibitors together can improve the function of immune cells to eliminate cancer cells. Thus, SCD1 and ACAT1 inhibitors can be used as drugs for cancer immunotherapy. Moreover, the SCD1 and ACAT1 pathways can be targeted to increase the effectiveness of immunotherapy.

https://onlinelibrary.wiley.com/doi/full/10.1111/CAS.15999

## Inhibition of TGF‐β signals suppresses tumor formation by regulation of tumor microenvironment networks

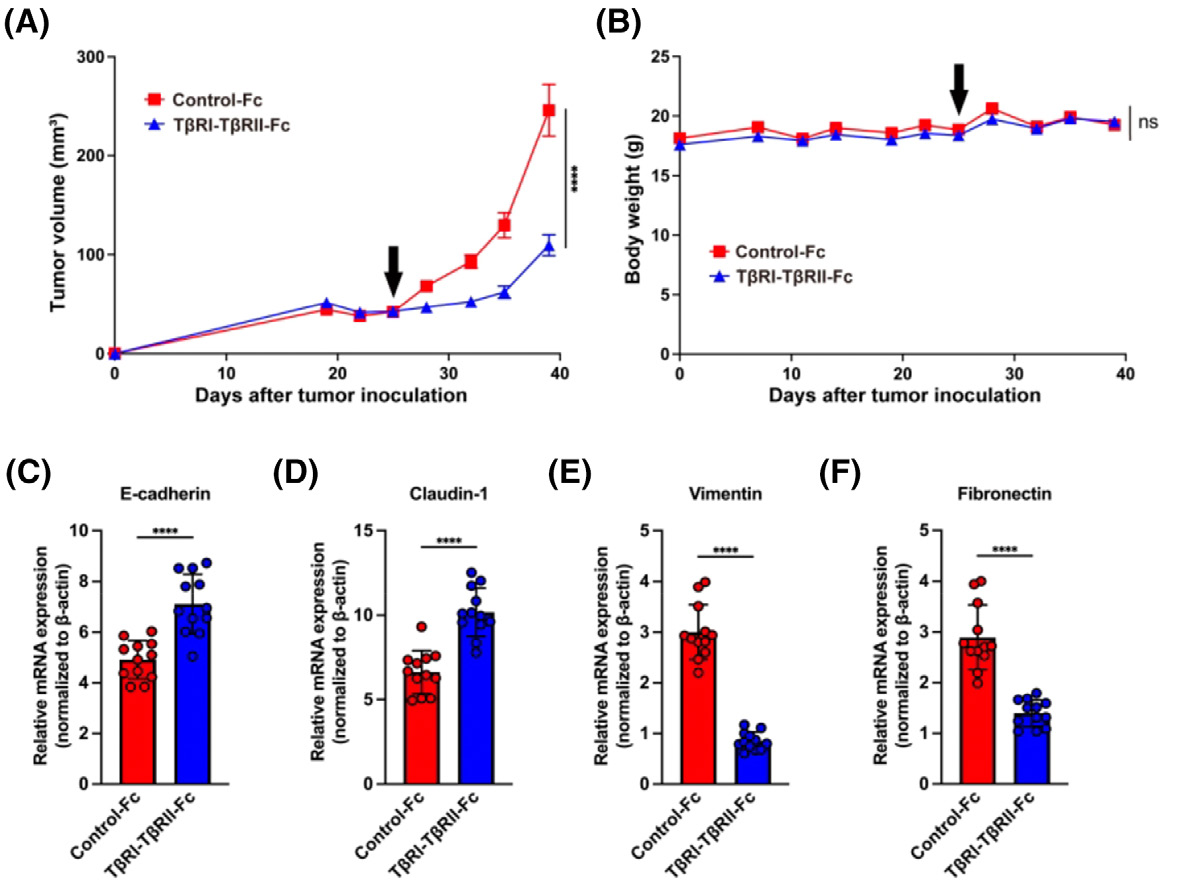

Cancer, disease characterized by cells that grow and divide in an uncoordinated, abnormal fashion, continues to have a very high global death rate despite the availability of multiple treatment strategies.

Many anticancer medications exhibit sub‐optimal effectiveness owing to the complexity of the tumor microenvironment (TME, a region surrounding the cancer cells). The TME consists of cancer cells, connective tissue called stroma, and numerous blood vessels, which collectively support tumor growth. The presence of transforming growth factor‐β (TGF‐β) in the TME, too, plays an important role in tumor growth.

TGF‐β stimulates tumor angiogenesis—the formation of blood vessels in the stroma—and facilitates epithelial–mesenchymal transition (EMT), a process that allows cancer cells to transition from being epithelial cells (or outer layer cells) into mesenchymal cells (which form the connective tissue), thus allowing them to migrate to other parts of the body and bolstering their metastatic potential.

In this study, Tokizaki et al. studied the anticancer activity of the TβRI‐TβRII‐Fc receptor protein they had previously developed. These receptor proteins can trap and block TGF‐β in the TME. Their findings showed that the TβRI‐TβRII‐Fc protein arrested EMT transition in lab‐cultured oral cancer cells. It also effectively suppressed tumor growth in mice.

The researchers attributed the mechanistic anticancer activity of the TβRI‐TβRII‐Fc protein to its ability to disrupt the production of regulatory proteins in the TME. These proteins—which include heparin‐binding epidermal growth factor‐like growth factor (HB‐EGF), interleukin‐1β (IL‐1β), and epiregulin (EREG)—help cancer cells divide uncontrollably and increase blood vessel formation to support their growth.

In summary, introduction of TβRI‐TβRII‐Fc receptors disrupts TGF‐β signaling in the TME by deactivating the HB‐EGF/IL‐1β/EREG pathways. This suggests that the TβRI‐TβRII‐Fc protein could potentially be used to target the TME of cancer cells and improve the treatment outcomes for patients with cancer.

https://doi.org/10.1111/cas.16006 Not published yet

